# Risk factors for diabetic retinopathy: a case–control study

**DOI:** 10.1186/s40942-016-0047-6

**Published:** 2016-09-12

**Authors:** Vinícius Carriero Lima, Gabriela Coutinho Cavalieri, Maurício Carriero Lima, Nazaré Otília Nazario, Gina Carriero Lima

**Affiliations:** 1 University of South Santa Catarina (UNISUL), Florianópolis, Santa Catarina Brazil; 2 University of South Santa Catarina (UNISUL), Av. José Acácio Moreira, 787, Tubarão, Santa Catarina Brazil

**Keywords:** Diabetic retinopathy, Risk factors, Diabetes complications, Diabetes mellitus

## Abstract

**Background:**

Diabetic retinopathy (DR) is the major cause of blindness among working age adults. The aim of the study was to investigate risk factors for development of DR.

**Methods:**

A case–control study was performed based on data from 240 individuals (80 cases and 160 controls) attending the Outpatient Specialty Clinic of the University of South Santa Catarina (UNISUL), between Mar/2010 and May/2014. Data collection occurred through review of medical charts for presence or absence of DR, determined by an ophthalmologist. Study protocol included demographic characteristics, metabolic control, diabetes mellitus (DM) profile and comorbidities. Statistical analysis used Chi square test for qualitative variables and multivariate logistic regression analysis to select independent variables (SPSS^®^18.0 software). Odds ratio (OR) was used as measure of association. The study was approved by research ethics committee of UNISUL.

**Results:**

Mean age of group case was 59.5 years with a slight female predominance. Gender, age, body mass index were not associated with outcome. Individuals with poor glycemic control were more likely to DR (OR 3.83; 95 % CI 1.57–9.37). It was observed a positive relationship between duration of DM and DR, with higher chances in 11–15 years of disease (OR 7.52, 95 % CI 3.03–18.68) and >15 years (OR 9.01, 95 % CI 3.58–22.66). Regarding comorbidities, only diabetic nephropathy showed higher chance for DR (OR 3.32; 95 % CI 1.62–6.79).

**Conclusions:**

Diabetic patients after 10 years of disease with poor glycemic control and nephropathy have a higher chance of DR.

## Background

Worldwide, diabetic retinopathy (DR) is the leading cause of blindness among working age adults [1]. Estimated prevalence remains around 34.6 % (approximately 93 million individuals) and 10.2 % have an advanced stage of the disease [2]. The basic mechanisms by which diabetes mellitus (DM) generates microvascular complications are not fully elucidated. In fact, DR is a multifactorial disease and some studies reports the role of proinflammatory cytokines and angiogenesis stimulatory molecules in the pathogenesis of the disease, in addition to chronic inflammation and oxidative stress caused by leukocytes [3, 4].

Under normal conditions, retinal capillaries have mural cells, known as pericytes, which are responsible for the regulation of vascular caliber and control of the blood flow of retinal microcirculation. These cells found to be important in the disease development, since they are lost in the early stages of DR [5–7]. However, not only pericytes are affected by the disease. DR involves all of the retinal cellular elements, including glial cells, neurons, bipolar cells, amacrine cells and glanglion cells [8].

Recent studies reported that early signs of neuroretinal damage may even precede vascular signs of DR [9–13]. Rodrigues et al. [12], using spectral-domain optical coherence tomography (SD-OCT), demonstrated thinning in thickness of retinal nerve fiber layer in patients with DM without DR compared to controls. Nevertheless, the clinical significance of these outcomes should be investigated and whether they represent a target in diabetes treatment to prevent DR [12, 13].

Another key point for the development of vascular lesions is the capillary basement membrane thickening as a result of hyperglycemia, increased synthesis of basement membrane components and other factors. This thickening of the basement membrane is related to excess vascular permeability that leads to leaky vessels, compromised tight-junctions and increased vesicular transport [14]. Additionally, the role of genetic factors and their relationship with the pathogenesis of DR are being extensively studied, including the epigenetic mechanisms such as DNA methylation, histone modifications in chromatin and noncoding RNAs [15].

Although major risk factors—hyperglycemia and hypertension—are extensively studied and show a strong association with DR [16, 17], there are variations in consistency and pattern of these factors [2]. Some studies indicate that prolonged DM duration is indeed a well-established risk factor for DR [18–20]. Regarding microalbuminuria, studies report its association as a marker of microvascular dysfunction and DR [21, 22], however further studies are needed to confirm this relationship [21]. Other risk factors such as body mass index (BMI) show controversies in relation to its association with this disease [23]. The aim of this study was to investigate risk factors for DR.

## Methods

A case–control study was performed at the Outpatient Specialty Clinic of the University of South Santa Catarina (UNISUL) in Tubarão city, located in southern Brazil. Medical records of diabetic patients seen between March 2010 and May 2014 were analyzed. Cases and controls corresponded to recordings of patients with DR and without DR, respectively. The control group sample was calculated by OpenEpi^®^ (Open Source Epidemiologic Statistics for Public Health Version 2.3.1) with a 95 % confidence interval, power of 80 %, ratio of controls to cases: 2:1 and percentage of controls exposed to poor glycemic control: 46 % [24] and Odds ratio 2.23 [19]. Added 10 % for losses, sample calculation resulted in 86 cases and 170 controls.

Data was collected using a questionnaire especially developed for this research and included: presence or absence of DR, demographic data (gender and age), metabolic control (BMI and glycemic control), DM profile (diabetes duration, type of DM and insulin use) and comorbidities (hypertension, dyslipidemia, and diabetic nephropathy). Clinical charts were first selected for the presence or absence of DR, determined by an ophthalmologist according to clinical findings confirmed by direct and indirect ophthalmoscopy following the classification of the international clinical diabetic retinopathy disease severity scale [25]. This study included records of all diabetic patients attended in the study period and they were all examined by an ophthalmologist. Patients with lens opacities and those in which the pupil dilation could not be performed were excluded (pregnant, uncontrolled hypertension and suspected of narrow-angle glaucoma). All information was obtained from clinical charts and finally analyzed for the study variables.

Mean age was used as cutoff point, since the sample showed a normal distribution. It was established the target for glycemic control the glycated hemoglobin (HbA1c) ≤7.0 % [26]. Regarding comorbidities, diagnosis was entirely based on clinical charts, considering previous clinical history and laboratory tests. Hypertension was set as blood pressure ≥140/90 mmHg (in distinct measures during the outpatient care) or use of antihypertensive therapy [26]; dyslipidemia set as triglycerides ≥150 mg/dL and total cholesterol ≥200 mg/dL or use of statins for dyslipidemia [16]; diabetic nephropathy set as microalbuminuria 30–300 mg/L, macroalbuminuria >300 mg/L or previous diagnosis of disease [26].

Statistical analysis was performed by a commercially available statistical software package (SPSS for Windows, version 18.0, SPSS, Chicago, IL, USA) and involved construction of frequency distributions, verifying the presence of association between variables and outcome, using Chi square test for qualitative variables. Multivariate analysis was performed using logistic regression and the model included variables that showed effect with p < 0.20 in bivariate analysis, except for gender and age that are classically part of models. The significance was calculated using Wald test and Odds ratio (OR) was used as the measure of association (p < 0.05). This study was based on the Resolution 466/12 of the National Health Council and was approved by the Ethical Committee of UNISUL. Researchers declare no conflicts of interest.

## Results

A total of 240 medical records were included, 80 cases and 160 controls. Eighteen records were excluded; 14 for incomplete data associated to variables of interest and 04 charts for impossibility of fundoscopy due to cataract.

Descriptive analysis resulted in average age of 59.5 years for cases and 59.3 years for controls. The percentage of female patients was 63.75 % in both groups. The age group >60 years was observed in 51.25 % of cases and 48.12 % of controls.

Gender, age and BMI were not associated with higher chances of DR. Concerning glycemic control, it was observed that individuals with poor glycemic control had a chance of 3.83 (95 % CI 1.57–9.37) for DR (Table 1). Similarly, diabetes duration was significantly associated with development of DR. It was observed a positive relationship between duration of DM and DR, with higher chances in 11–15 years of disease (OR 7.52, 95 % CI 3.03–18.68) and >15 years (OR 9.01, 95 % CI 3.58–22.66) (Table 1). Regarding type of diabetes, our results demonstrated a chance of 2.37 (95 % CI 0.52–10.68) for DR in type 1 DM, but this association did not reach statistical significance (p = 0.261) (Table 1).

**Table 1:** Cases and controls according to demographic characteristics, metabolic control and diabetes mellitus profile

	Total (240)	Diabetic retinopathy				OR^b^	CI^b†^	OR^Adj^	CI^Adj‡^
		Yes (80)		No (160)					
	n	n	%	n	%				
Gender									
Female	153	51	33.3	102	66.7	1.00	0.57–1.74	1.13	0.57–2.26
Male	87	29	33.3	58	66.7	1		1	
Age (years)									
≤60	122	39	32.0	83	68.0	1		1	
>60	118	41	34.7	77	65.3	1.13	0.66–1.93	1.28	0.64–2.56
BMI									
18.5–24.9	39	13	33.3	26	66.7	1.09	0.51–2.35	–	–
25–30	70	26	37.1	44	62.9	1.29	0.70–2.38	–	–
>30	131	41	31.3	90	68.7	1		–	–
Glycemic control (%)									
HbA1c ≤ 7	80	10	12.5	70	87.5	1		1	
HbA1c > 7	160	70	43.8	90	56.3	5.43**	2.61–11.36	3.83*	1.57–9.37
Diabetes duration (years)									
<5	87	14	16.1	73	83.9	1		1	
6–10	67	14	20.9	53	79.1	1.37	0.61–3.13	2.05	0.71–5.92
11–15	26	12	46.2	14	53.8	4.46*	1.71–11.63	7.52**	3.03–18.68
>15	60	40	66.7	20	33.3	10.42**	4.76–22.73	9.01**	3.58–22.66
Type of diabetes									
Type 1	11	6	54.5	5	45.5	2.51	0.74–8.50	2.37	0.52–10.68
Type 2	229	74	32.3	155	67.7	1		1	
Insuline use									
Yes	112	52	46.4	60	53.6	3.09**	1.76–5.41	1.25	0.61–2.57
No	128	28	21.9	100	78.1	1		1	

OR^b^: *Odds*-*ratio* Brut, OR^Adj^: adjusted *Odds*-*ratio*

* *p* < 0.05; ** *p* < 0.01 (Pearson’s Chi square test and Wald test)

^†^95 % of confidence interval Brut; ^‡^ 95 % adjusted confidence interval

In bivariate analysis, it was found association between DR and HbA1c >7 %, duration of DM ≥11 years and insulin use. After logistic regression, insulin use lost association, while other variables (glycemic control and duration of DM) remained associated with DR, regardless other variables included in the model (Table 1).

Multivariate analysis of comorbidities showed a chance to DR in patients with diabetic nephropathy of 3.32 (95 % CI 1.62–6.79) (Table 2). Other comorbidities studied, hypertension and dyslipidemia, did not reach the statistical significance level established (p < 0.05).

**Table 2:** Cases and controls according to comorbidities

	Total (260)	Diabetic retinopathy				CI^b†^	OR^b^	CI^Adj‡^	OR^Adj^
		Yes (80)		No (160)					
	n	n	%	n	%				
Hypertension									
Yes	204	71	34.8	133	65.2	1.60	0.71–3.59	–	–
No	36	9	25.0	27	75.0	1		–	–
Dyslipidemia									
Yes	176	66	35.8	112	64.2	1.54	0.81–2.90	1.41	(0.61–3.25)
No	64	17	26.6	47	73.4	1		–	–
Diabetic nephropathy									
Yes	69	39	56.5	30	43.5	4.12**	2.28–7.44	3.32*	(1.62–6.79)
No	171	41	24.0	130	76.0	1			

OR^b^: *Odds*-*ratio* Brut, OR^Adj^: adjusted *Odds*-*ratio*

* *p* < 0.05; ** *p* < 0.01 (Pearson’s Chi square test and Wald test)

^†^95 % of confidence interval Brut; ^‡^ 95 % adjusted confidence interval

## Discussion

Descriptive analysis of demographic characteristics in both groups presented a similar mean age (59.5 vs 59.3 years, cases and controls respectively) with a slight predominance of females. In qualitative analysis, the variables age and gender were not associated with a greater chance of DR. Similarly, in several studies, no association was found between DR and gender, age or age group [27–31]. However, some reports have found an independent association in males [18, 32, 33], while a historical cohort of Kajiwara et al. [34], presented greater chance of DR among females. Yet, in disagreement with the present study, Al-Sarraf et al. [35] observed a greater chance of DR in individuals aged 50–59 years (OR 2.2, 95 % CI 1.1–5.2) and ≥60 years (OR 4.6, 95 % CI 11.0–2.0). Present findings show that probably age and gender are not risk factors for DR. Therefore, regular monitoring of these patients must be done regardless these variables.

Among metabolic control factors, none of BMI categories revealed higher chances to DR. Literature differs in this question and demonstrates a controversy with this association, especially about type 2 DM [23]. Several studies show no association between BMI and DR [18, 21, 31, 36–39] and other positive association [34, 40, 41]. Ahmed et al. [28], in a follow-up of involving 977 type 2 DM during 5, 10 and 15 years, did not find an increased risk of DR in any of categories studied. Yoshida et al. [42] presented statistical significance in DR frequencies in patients with BMI > 23.7 kg/m^2^ only after 7 years of follow-up. Our findings appear to corroborate the results reported in other studies and BMI does not seem to have direct relationship with development of DR.

Regarding glycemic control, individuals with HbA1c >7 % were more likely to develop DR. Chronic hyperglycemia is responsible for a chain of events that leads to DR [43] and glycemic control one of the key points of primary prevention for this complication [29, 44, 45]. Strong evidences indicate an adequate glycemic control in levels of HbA1c ≤7 % as a reducer of risk for DR, both in type 1 DM and in type 2 DM [32, 46, 47]. Accordingly to a longitudinal study [28], there was an association between DR and poor glycemic control showing higher OR for intervals of 5, 10 and 15 years of 8.34, 3.45 and 4.9, respectively. In the present study, inadequate control of blood glucose levels proved to be an important factor for DR. After diagnosis of DM, patients must reach the targets for control of glycemia, reducing the chances of visual impairment.

Duration of DM, an important predictor DR, determines the exposure time of other risk factors [48]. In the current study, after 10 years of disease, results showed high association. Al-Sarraf et al. [35] noticed that patients with 10–19 years of disease are twice as likely to have DR and approximately three times more when the exposure is more than 20 years. According to a recent study, conducted in United States of America [18], each year of DM represents a 6 % increase in chance of DR. This correlation with duration of diabetes was first demonstrated in the Wisconsin epidemiologic study of diabetic retinopathy (WESDR) [49], with a higher prevalence in 25, 60 and 80 % for 5, 10 and 15 years of evolution of DM, respectively. In addition to aforementioned, several other studies, show statistical significance for this factor, both in type 1 DM and in type 2 DM [27, 29, 30, 36, 37], perhaps the most important independent risk factor for DR [31, 50, 51].

Type of DM was not considered a risk factor for DR. Similarly, in a multicenter study in Kuwait, Al Sarraf et al. [35] found no significant results when comparing same variables. However, WESDR, in 25 years of follow-up of type 1 DM subjects, demonstrated cumulative incidence rates of 97 % for DR [52]. The authors suggest that the lack of association probably occurred due to small number of DM type 1 individuals in the study’s sample.

Concerning insulin therapy, an interesting finding was observed. In bivariate analysis, patients using insulin presented a highly significant chance of DR. In multivariate analysis, odds for DR lost statistical significance and strength of association. Furthermore, Yang et al. [29] found no statistically significant after multivariate analysis (OR 1.65, 95 % CI 0.80–3.38). On the other hand, some studies have shown significantly higher chances in diabetics treated with insulin, OR 3.23 [21] and OR 3.51 [8], but only included individuals aged above 40 and 67 years, respectively. One of the most important studies on the subject, the United Kingdom Prospective Diabetes Study [47], suggested that adequate and continuous glycemic control inhibits development of DR, regardless the method of treatment. Other major study, the Diabetes Control and Complications Trial (DCCT), found that intensive insulin treatment reduces the risk of development of DR and slows the progression of clinically important retinopathy. In patients without retinopathy at enrollment, the 3-year risk of developing retinopathy was reduced by 75 % in the intensive insulin treatment group compared to the conventional treatment group [53].

In the present study, the authors suggest that probably the absence of significance between insulin use and DR occurred as a reflection of reverse causality bias. In a similar way, the WESDR observed no higher relative risk for DR with insulin use among patients >30 years [49]. Yoshida et al. [42], found that change of therapy method modifies incidence rate of DR and argued that individuals under insulin therapy experience a more severe metabolic deterioration, not being probably insulin use an isolated risk factor for this diabetic complication.

About blood pressure, even in absence of established hypertension, changes in homeostasis of blood pressure levels appear to be associated with DR [23]. In a study with diabetic over 67 years [21], the association between higher levels of systolic pressure and DR at any stage occurred, but in agreement with the present study, hypertension as a comorbidity was not an independent risk factor for DR, as well as in other studies [29, 31, 36, 54]. Nevertheless, Zheng [54] concluded that the relationship between these two variables cannot be excluded, since isolated pressure measurements may not accurately represent continued effect of high pressure. In agreement, some studies indicate hypertension as one of the most important risk factors in the development of DR [50, 51, 55, 56].

Many researchers have shown that the relationship between serum lipids and DR does not seem consistent or loses association after adjustment for confounding factors [27–30, 37]. The Beijing Eye Study [36] reported no association between increased cholesterol levels and DR. Thus, lipid changes do not appear to be the main responsible for microvascular involvement. Even though, in the current study, dyslipidemia have not been associated with DR, the importance of diet quality of patients cannot be underestimated. Moreover, in addition to glycemic control, HbA1c and lipid profile investigations are recommended to continue as routine when we are handling these patients [57].

Microvascular complications such as diabetic neuropathy and DR can be correlated and they usually follow with the onset of diabetic nephropathy, which suggests a strong association between them [23, 58]. Jost et al. [27], after logistic regression revealed a chance of 3.24 (95 % CI 1.04–10.04) for DR in patients with diabetic nephropathy. Rajalakshmi et al. [37] observed a significant presence of microalbuminuria in both type 1 DM and type 2 DM individuals with early diagnosis of DM (p = 0.001 and p = 0.009, respectively). Studies indicate that the damage to the small blood vessels share the same pathogenesis, part of a systemic microvascular dysfunction [59, 60].

The strengths of this study allowed the validation of results obtained. First, information sources showed an excellent quality because patient records were complete and properly filled. Second, it is an outpatient specialty clinic with adequate infrastructure for care and for monitoring of patients that maximizes the formation of representative groups of cases and controls. Third, we undertook methodological efforts to enable scientific comparability using multivariate data analysis in order to reduce confusing factors. Fourth, it highlights a significant population problem and there is no major epidemiological study in Brazil about this topic. Therefore, our study amplifies the comparability of data regarding the reality of DR in Brazil.

In addition to its strengths, there are some limitations in the present study. It was not possible to perform more accurate tests, such as fluorescein angiography or SD-OCT, for all cases and controls. Consequently, some patients with DR could have been inadvertently excluded from this study. Moreover, since it is a chart review criteria of time of disease may have been inaccurate, however considering the fact that these patients had a longer time of disease before the diagnoses it only generates an underestimation of data. Lastly, we could not estimate specific OR for each variable of individuals with type 1 and type 2 DM individually, since the sample was mainly composed of type 2 DM individuals.

## Conclusions

According to our results, diabetic patients after 10 years of disease with poor glycemic control and nephropathy have a greater chance of DR. The mechanisms underlying DR development are not fully elucidated. Thus, the scientific publication in this area should intensify, especially in Brazil, to improve prevention, identify risk groups and establish real modifiable factors of this highly prevalent disease.

## Abbreviations

BMI: body mass index
CI: confidence interval
DM: diabetes mellaitus
DR: diabetic retinopathy
HbA1c: glycated hemoglobin
OR: odds ratio
SD-OCT: spectral-domain optical coherence tomography
UNISUL: University of South Santa Catarina
WESDR: Wisconsin epidemiologic study of diabetic retinopathy
